# Carryover effects across juvenile periods vary by species, cause-specific mortality, and stage-specific behavior

**DOI:** 10.1007/s00442-026-05887-5

**Published:** 2026-06-19

**Authors:** Emily R. Shertzer, Donald W. Jones, Anna D. Chalfoun

**Affiliations:** 1 Wyoming Cooperative Fish and Wildlife Research Unit, Department of Zoology and Physiology and Program in Ecology and Evolution, University of Wyoming, Laramie, WY USA; 2 Wyoming Cooperative Fish and Wildlife Research Unit, Department of Zoology and Physiology, University of Wyoming, Laramie, WY USA; 3 United States Geological Survey Wyoming Cooperative Fish and Wildlife Research Unit, Department of Zoology and Physiology and Program in Ecology and Evolution, University of Wyoming, Laramie, WY USA

**Keywords:** Weather-related mortality, Predation-related mortality, Sympatric species, Sagebrush steppe, Hazards models

## Abstract

Conditions during one life-stage can carry over to influence fitness outcomes in later stages. How carryover effects manifest, however, may vary depending on the type and magnitude of risks that animals face in subsequent life-stages. The influence of carryover effects on cause-specific mortality remains largely unclear, particularly during vulnerable life-stages, including juvenile periods. We proposed and investigated the *Cause-specific carryover hypothesis,* that the relative influence of traits developed during a previous stage on mortality during the next vary by cause of mortality, using juvenile sagebrush songbirds (Brewer’s sparrow, *Spizella breweri*; sagebrush sparrow, *Artemisiospiza nevadensis*; sage thrasher, *Oreoscoptes montanus*) as a focal system. We tracked birds during the post-fledging period (a juvenile period following the nestling period), identified the rates and causes of mortality, and tested the relative influence of different nestling traits (wing length, tarsus length, feather development, and body condition) on post-fledging mortality risk. Carryover effects varied across co-occurring species and differed by cause of mortality, and tarsus length had an unexpected and outsized impact on predation risk in the two sparrows. Although wing morphology typically is considered the most important trait for mobility in birds, most mortalities occurred during the first 5 days of the post-fledging period when altricial fledglings were incapable of flight and likely relied on running or scrambling ability to escape predators. Therefore, carryover effects were driven by stage-specific behavior during the period of highest risk. Developing animals may face investment trade-offs between morphological traits that influence different types of risk during different subsequent stages.

**Supplementary Information:**

The online version contains supplementary material available at 10.1007/s00442-026-05887-5.

## Introduction

Organisms often experience different selective pressures and vital rates across life-stages, which has important implications for population dynamics (Berger et al. 2021; Low and Part 2009; Sillett and Holmes 2002; Sniegula et al. 2017). Early life-stages in particular often are characterized by high rates of mortality because of the heightened predation risk or environmental hazards specific to juveniles (Laurenson 1994; Polte et al. 2014). Juveniles may be more susceptible than adults to predation, for example, because of smaller body size or decreased mobility (McLennan et al. 2004; Aastrup et al. 2023). Identifying the factors influencing mortality during early life-stages therefore can be important for understanding population and community dynamics (Kanno et al. 2015; Sergio et al. 2021; Keevil et al. 2023).

Environmental conditions during development may not cause immediate mortality, but still can influence survival or performance in later life-stages (Norris 2005; Harrison et al. 2011; Moore and Martin 2019; Cooper et al. 2024). Whereas examples of such carryover effects exist for some species and life-stages, the prevalence of carryover effects (particularly between vulnerable juvenile periods) and how they vary across co-occurring species remains unclear (O’Connor et al. 2014; Marra et al. 2015; Lundsgaard et al. 2023). Whether and how carryover effects manifest likely depend upon the types of risks that organisms face in subsequent life-stages and the traits that help buffer against different stressors. For example, poor body condition in fledgling dickcissels (*Spiza americana*) correlated with higher mortality during extreme weather events, whereas small wings were associated with higher risk of predation (Jones et al. 2017). Investigation of carryover effects on cause-specific mortality risk remains rare, however, partially because of the challenges of ascribing causation of mortality post hoc.

Birds are an ideal group with which to study carryover effects across juvenile stages because young birds navigate multiple and distinct stages, each with potentially discrete rates of associated risk (Martin et al. 2018). Many avian species experience survival bottlenecks after leaving the nest prior to independence. Post-fledging mortality of songbirds, for example, can reach 70% during the first 3 weeks (Suedkamp Wells et al. 2007; Cox et al. 2014). Differences in risk and mortality rates between the nestling and post-fledging periods, however, remain unknown for most species. Furthermore, cause-specific mortality and associated carryover effects rarely are identified in post-fledging studies (Suedkamp Wells et al. 2007; Jones et al. 2017). Examination of carryover effects between the nestling and post-fledging period typically has focused on specific traits, especially wing development and various measures of body condition (Vitz and Rodewald 2011; Jones et al. 2017; Martin et al. 2018; Jones and Ward 2020). The focus on wing development in particular stemmed from evidence that wing morphology can influence predator evasion and dispersal ability (Dial et al. 2006; Weeks et al. 2022). The most influential traits for survival, however, may vary depending on environmental context, predominant risks, and evolved behavioral strategies. For example, fledglings of many avian species cannot fly within the first few days post-fledging, raising the possibility that traits enhancing locomotion on the ground may be more critical than those improving flight performance (Ducatez and Field 2021). Furthermore, even among co-occurring species that share the same environment, the predominant risks for fledglings may vary as species differ in their morphology or behavior (Sinclair 2003; Jones et al. 2026).

We evaluated whether carryover effects from the nestling to the post-fledging period varied among co-occurring taxa or according to stage-specific risk and identified the traits of nestlings most associated with survival during the post-fledging period. We tested carryover effects in three sympatric species of passerines (Brewer’s sparrow, *Spizella breweri*; sagebrush sparrow, *Artemisiospiza nevadensis*; and sage thrasher, *Oreoscoptes montanus*) that breed within North American sagebrush steppe, an open, arid-land ecosystem where songbirds face highly variable weather regimes and numerous potential predators. All three species build open-cup nests in sagebrush shrubs (*Artemisia* spp.), with nest failures typically caused by predation or extreme weather (Hethcoat and Chalfoun 2015a; Hightower et al. 2018). The three species, however, vary in body mass (Brewer’s sparrow = 10.6 ± 3.9 SD g; sagebrush sparrow = 19.1 ± 1.4 SD g; sage thrasher = 39.0 ± 5.6 SD g; Shertzer and Chalfoun unpublished data) and the length of the nestling period (Brewer’s sparrow = 7–9 days; sagebrush sparrow = 8–10 days; sage thrasher = 10–12 days). The focal system therefore facilitated investigation of carryover effects across species that shared habitat and risks yet varied in their morphological and life-history traits.

We quantified mortality rates during the nestling and post-fledging periods and identified the proximate causes of mortality during the post-fledging period as predation or weather-related exposure. We then examined how development of traits during the nestling period carried over to influence post-fledging survival, and which traits were most influential for each species. Finally, we proposed and investigated the *Cause-specific carryover hypothesis* that the relative influence of traits developed during a previous stage on mortality during the next would vary by cause of mortality. We predicted that more developed wings or tarsi in nestlings would reduce post-fledging predation through enhanced mobility. Accordingly, we evaluated the assumption that individuals that fledged with more developed wings would demonstrate the ability to fly at a younger age. By contrast, we predicted that better body condition and feather development of nestlings would reduce weather-related mortality during the post-fledging period because of the thermoregulatory benefits, particularly for the smaller birds (Deville et al. 2014; Terrill and Shultz 2023). Finally, we tested the associated assumption that weather-related mortalities should be more prevalent following extreme weather.

## Materials and methods

### Study area

We conducted our study within sagebrush-steppe habitat in Sublette County, Wyoming USA (42°38’ N, 109°45’ W) at six 25-ha sites established in 2008 and 2011 (Gilbert and Chalfoun 2011; Hethcoat and Chalfoun 2015a). Sites were distributed across an approximately 360 km^2^ study area, separated by ≥ 1 km, and spanned a gradient of surrounding surface disturbance associated with natural gas development. The elevation of the study area ranged from 2100 to 2400 m. Temperatures during the study period ranged from − 4 to 35 ˚C. Precipitation events tended to be sporadic and included snow, hail, and thunderstorms.

### Nestling measurements

We located nests of Brewer’s sparrows, sagebrush sparrows, and sage thrashers during early-May to mid-August 2021–2023 using systematic searches and behavioral observations. We spent approximately equal nest-searching effort across all sites. Nests were monitored every 1–2 days until failure or fledging. For nests with known hatch dates, we affixed an aluminum band (U.S. Geological Survey), to identify individuals, and a plastic color-band, to visually identify broods, to the legs of nestlings on the day before feather eruption (5, 6, and 7 days after hatch for Brewer’s sparrows, sagebrush sparrows, and sage thrashers, respectively). We measured morphometrics of nestlings at the end of the nestling period to best represent traits integrated across the nestling period. Measurements occurred on the average day of fledging for each species, which was 8, 9, and 10 days after hatch for Brewer’s sparrows, sagebrush sparrows, and sage thrashers, respectively, to reduce the potential for negative effects of early force-fledging (Streby et al. 2013). We measured un-flattened wing chord length (hereafter, wing length) using a wing chord ruler (± 1 mm), manus (wrist joint to the end of the terminal phalanx), exposed culmen, tarsus, feather length (length of the longest primary feather), and plume length (length of the longest broken plume on a primary feather) using digital calipers (± 0.1 mm), and mass using a digital scale (± 0.1 g; Pyle 1997).

We assessed multiple indices of body condition, because selection of the most appropriate index for body condition may vary depending on the species or context (Schamber et al. 2009; Labocha and Hayes 2012). We performed a principal component analysis using all skeletal measurements (manus, culmen, and tarsus) and used the first component as a measure of structural size (Freeman and Jackson 1990). We then created four potential body condition indices: 1) the residuals from an ordinary least squares regression of structural size on body mass (Labocha and Hayes 2012), 2) a scaled mass index using mass and tarsus length (Peig and Green 2009), 3) the residuals of tarsus length regressed on body mass (Vitz and Rodewald 2011), and 4) mass alone. We selected the residuals of body mass regressed on structural size as the most appropriate measure of body condition for all three species, after comparing univariate Cox proportional hazards models (hereafter, Cox models) with body index as the only fixed effect using Akaike Information Criterion scores corrected for small sample size (AICc). Hereafter, we refer to this as body condition. We also created an index of body size to control for potential confounding effects of overall body size. The size index consisted of the first component from a principal components analysis that included manus, culmen, and mass, and was not correlated with the covariates of interest. Finally, we measured the longest primary feather and divided the length of the broken plume by the full feather length as a proxy for wing coverage.

### Fledgling tracking

We affixed radio tags to up to three randomly selected nestlings from broods of known ages. We used battery-powered UHF radio tags (PowerTag, 0.35 g, 434 MHz, Cellular Tracking Technologies; Rio Grande NJ, USA) and a leg-loop harness attachment (Streby et al. 2015). Harnesses were constructed of stretch jewelry cord (Dritz 9345B Elastic Thread, 0.35 mm). We tracked fledgling Brewer’s sparrows during 2021–2023, and fledgling sagebrush sparrows and sage thrashers during 2022–2023. We located fledglings using hand-held radio telemetry approximately every 1–3 days until mortality or dispersal from the site. We defined dispersal as at least 4 days without a signal detected within approximately 500 m of the fledgling’s previous location, based on field observations that fledglings were rarely detected after two to three relocation attempts with no signal. We noted the movement behavior of fledglings upon relocation (remained still, ran, flew weakly or flapped, or full flight). We defined movement as flight if the fledgling became airborne, even if only momentarily.

**Fig. 1:**
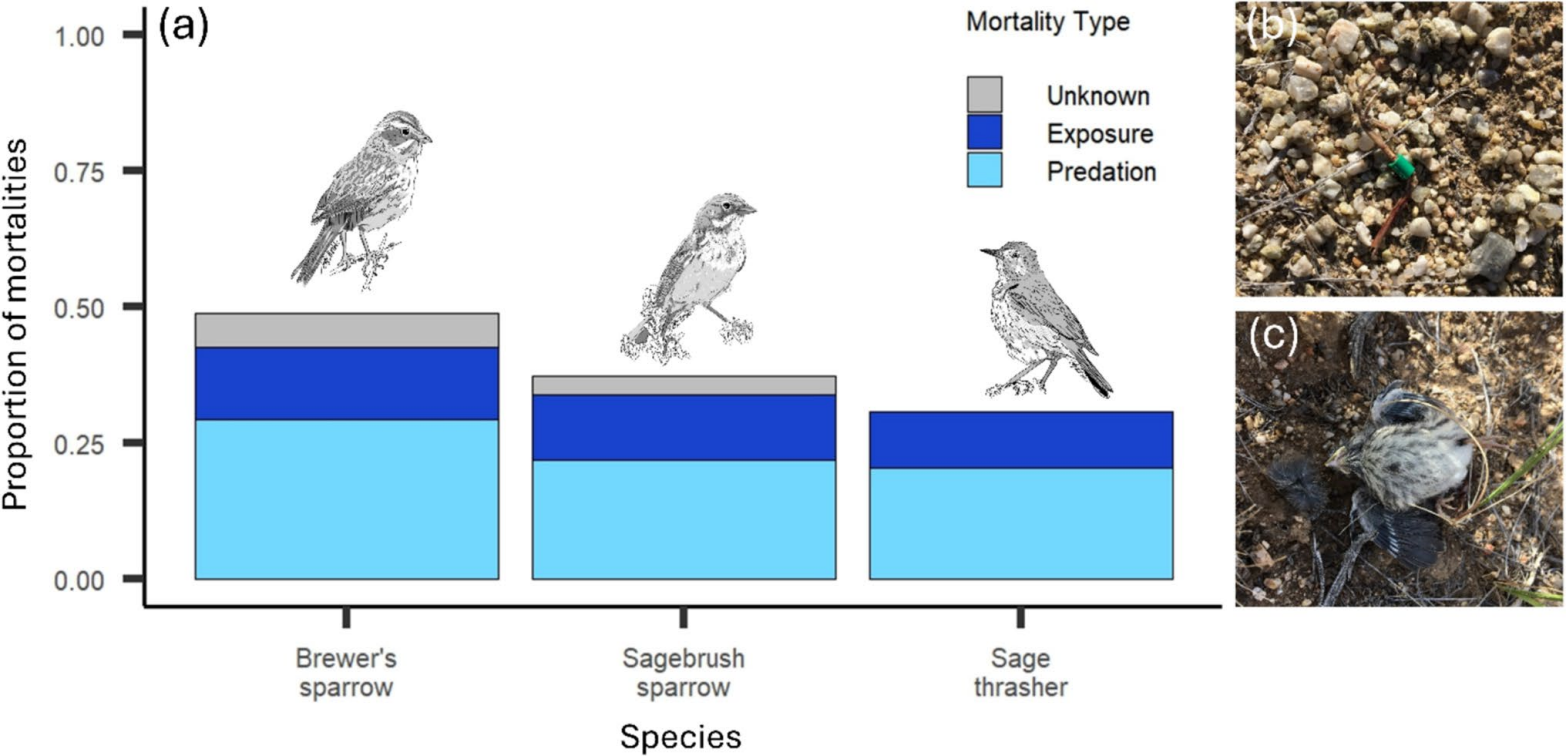
**a** The proportion of Brewer’s sparrow (*n*=129), sagebrush sparrow (*n*=59), and sage thrasher (*n*=68) fledglings tracked in Wyoming, USA during 2021–2023 that died during the post-fledging period varied between 0.31 and 0.49. Mortalities were attributed to unknown causes (grey), exposure (dark blue), or predation (light blue). **b** Signs of predation included dismembered limbs, large bite marks, or carcasses recovered in small mammal burrows, whereas **c** we assumed that intact carcasses died by exposure

When a mortality occurred, we classified the cause as either predation- or exposure-related. Fledglings that died of predation typically were dismembered, partially eaten, or recovered in small mammal burrows, whereas those that died of exposure were fully intact and undamaged (Fig 1). We classified the cause as unknown if a body was found with only a small wound, was too damaged by ants to identify a cause, or had not been relocated for several days and therefore might have been scavenged posthumously. We relocated individuals every 1–2 days during the early post-fledging period to reduce the possibility of misclassifying scavenged carcasses as depredated. Although we assumed that intact mortalities were primarily caused by exposure, other factors including disease, starvation, or parasites may have also contributed (Jones et al. 2026).

We extrapolated the minimum regional temperature recorded between relocations to test the assumption that exposure mortalities were coupled with weather extremes. Temperature data were collected at regional weather stations in the National Weather Service Cooperative Observer Program (National Oceanic and Atmospheric Administration 2024) within 28 km of the field sites. Temperature data for the northern sites were collected at hourly intervals in the town of Pinedale, WY (42°52’30” N, 109°51’44” W) and data for the southern sites were collected in Boulder, WY (42°42’55” N, 109°41’21” W). We calculated the mean and standard deviation of the minimum temperature fledglings experienced preceding live relocations and exposure-attributed mortalities.

### Statistical analyses

We conducted all analyses in Program R (R Core Team 2024). We calculated daily survival probability during the nestling and post-fledging periods from Kaplan–Meier curves using the package ‘survival’ (Therneau 2024). We only included nests found prior to or on the day of hatching. To test nestling period survival, we subset the period from hatch day to the first possible day of fledging.

We tested the effects of nestling morphology on mortality risk during the post-fledgling period using mixed-effects Cox models and the package ‘coxme’ (Therneau 2022). We included year and nest ID as nested random effects in all models. We initially included study site as a random effect, but because it had little effect, did not improve model fit, and spatial effects also were accounted for by the random effect of nest ID, we removed study site from final model suites. We included wing length, tarsus length, feather development, and body condition as fixed effects. We scaled and centered all covariates for comparison. We did not include covariates with a Pearson’s correlation coefficient of 0.7 or greater within the same model to prevent issues with collinearity, and we removed missing observations from models (up to 45 removed of 639 observations).

We constructed a mixed-effects Cox model with all data to compare risk across species. We then separated datasets by species to test carryover effects. For each species, we censored competing events to assess cause-specific hazards using the standard Cox regression (Andersen et al. 2012; Wolbers et al. 2014). We separated datasets into overall mortality risk (all fledglings tracked), predation risk (only fledglings that survived and fledglings that died because of predation while censoring exposure mortalities), and exposure risk (only fledglings that survived and fledglings that died from exposure while censoring predation mortalities).

To account for the possibility of bias between observers on nestling measurements (Barrett et al. 1989), we tested the effect of observer on measurements using analysis of variance tests and compared model suites with and without a random effect of observer. Observer had no effect and was not included in final models. To account for the potential effects of overall body size, we tested all model suites with and without a body size index. Overall body size did not influence model results and decreased model fit, so we did not include a body size index in the final model suite.

We checked the Schoenfeld residuals for the global Cox model of each species to test for non-proportional hazards (Grambsch and Therneau 1994). Schoenfeld residuals were insignificant for all covariates except body condition in sage thrashers (Online Resource Fig S1-3). We determined that the failure of the Schoenfeld residual test for body condition in sage thrashers occurred because of a low sample size of mortalities after the first day of the post-fledging period and did not affect the interpretation of the result (Therneau and Grambsch 2000).

We compared all hypothesized models using AICc scores for small sample sizes (Hurvich and Tsai 1989; Sugiura 1978; Online Resource Tables S1-7). We selected top models based on a ΔAICc < 2 (Burnham and Anderson 2002; Sutherland et al. 2023). The model averaged the top models if multiple top models fell within 2 ΔAICc using the package ‘MuMIn’ (Burnham and Anderson 2002; Bartoń 2023). All candidate models represented plausible a priori hypotheses, as combinations of nestling traits could affect risk from multiple causes.

We tested whether wing length influenced the timing of initiation of first flight with a generalized linear mixed model (GLMM) using the package ‘glmmTMB’ (Brooks et al. 2017). We included year and nest ID as nested random effects. We only tested flight initiation for Brewer’s sparrows, because behavioral differences among the three species made it difficult to determine flight initiation in sagebrush sparrows and sage thrashers. Unlike Brewer’s sparrows, sagebrush sparrow and sage thrasher adults and fledglings were frequently flushed by running rather than flying. As a result, we could not confidently distinguish whether running fledglings were truly incapable of flight (Online Resource Fig S4).

## Results

**Fig. 2:**
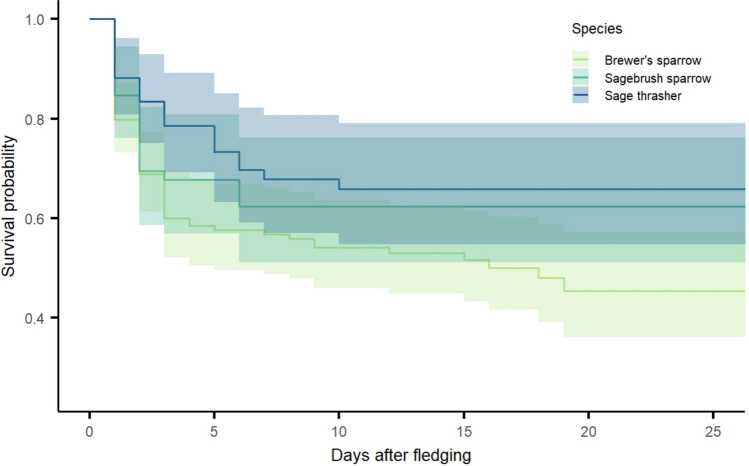
Survival decreased non-linearly over time after fledging for Brewer’s sparrows, sagebrush sparrows, and sage thrashers tracked in Wyoming, USA in 2021–2023. Survival curves were calculated using the Kaplan–Meier estimator and 95% CI

We tracked juveniles for up to 34 days after fledging. Survival rates during the post-fledging period were lower than those during the nestling period (Table 1). The overall risk of mortality during the post-fledging period was similar for the two sparrow species, but approximately twice as high for the smallest (Brewer’s sparrow) compared with the largest (sage thrasher; Hazard Ratio = 0.54) species (*n* = 105 events, *β* = − 0.61 ± 0.28 SE, *P* = 0.031) (Fig 1, Online Resource Table S8). Similarly, the risk of predation during the post-fledging period was marginally higher for Brewer’s sparrows compared with sage thrashers (Hazard Ratio = 0.55, *n* = 65 events, *β* = − 0.60 ± 0.33 SE, *P* = 0.070) but did not differ between the two sparrows. Exposure risk did not vary by species. Most mortalities (86%, 86%, and 81% of Brewer’s sparrow, sagebrush sparrow, and sage thrasher, respectively) occurred within the first 5 days of the post-fledging period (Fig 2). Indeed, the most likely time for mortality to occur was during the first 24 h out of the nest (41% of mortalities for both sparrows and 38% of sage thrasher mortalities).

**Table 1:** Nestling survival to the earliest possible fledging age (7, 8, and 9 days after hatching for Brewer’s sparrows, sagebrush sparrows, and sage thrashers, respectively) was greater than fledgling survival over the same temporal extent (e.g., 7, 8, and 9 days after leaving the nest for Brewer’s sparrows, sagebrush sparrows, and sage thrashers, respectively) in Wyoming, USA during 2021–2023 (Brewer’s sparrows) and 2022–2023 (sagebrush sparrows and sage thrashers)

Species	Nestling period				Fledgling period			
	*n*	Survival estimate	SE	95% CI	*n*	Survival estimate	SE	95% CI
Brewer’s sparrow	2526	0.79	0.02	0.75–0.82	129	0.57	0.04	0.49–0.67
Sagebrush sparrow	988	0.89	0.02	0.85–0.92	59	0.62	0.06	0.51–0.76
Sage thrasher	876	0.88	0.02	0.84–0.92	68	0.68	0.06	0.57–0.81

Survival estimates were calculated using Kaplan–Meier curves. A table containing the full Kaplan–Meier curve with daily survival estimates is included in the Electronic Supplemental Material (Online Resource Table S9)

### Carryover effects

**Fig. 3:**
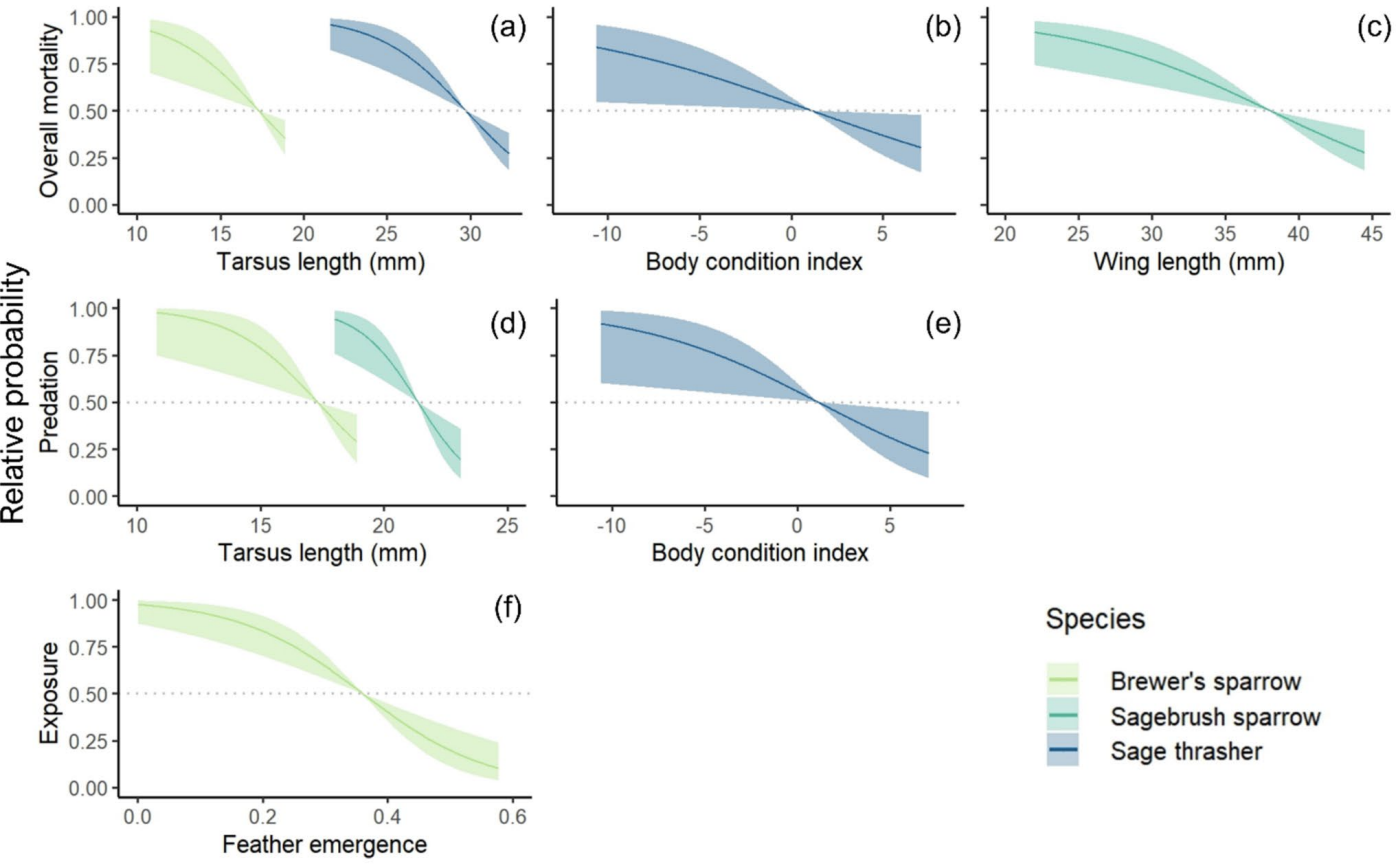
Tarsus length, body condition, wing length, and feather emergence influenced the probability of **a**–**c** overall mortality, **d**–**e** predation-caused mortality, and **f** exposure-caused mortality relative to the population average (indicated with the dotted line) in Brewer’s sparrows, sagebrush sparrows, and sage thrashers tracked during the post-fledging period in Wyoming, USA in 2021–2023. Predictions from top Cox models with 95% CI are shown

**Fig. 4:**
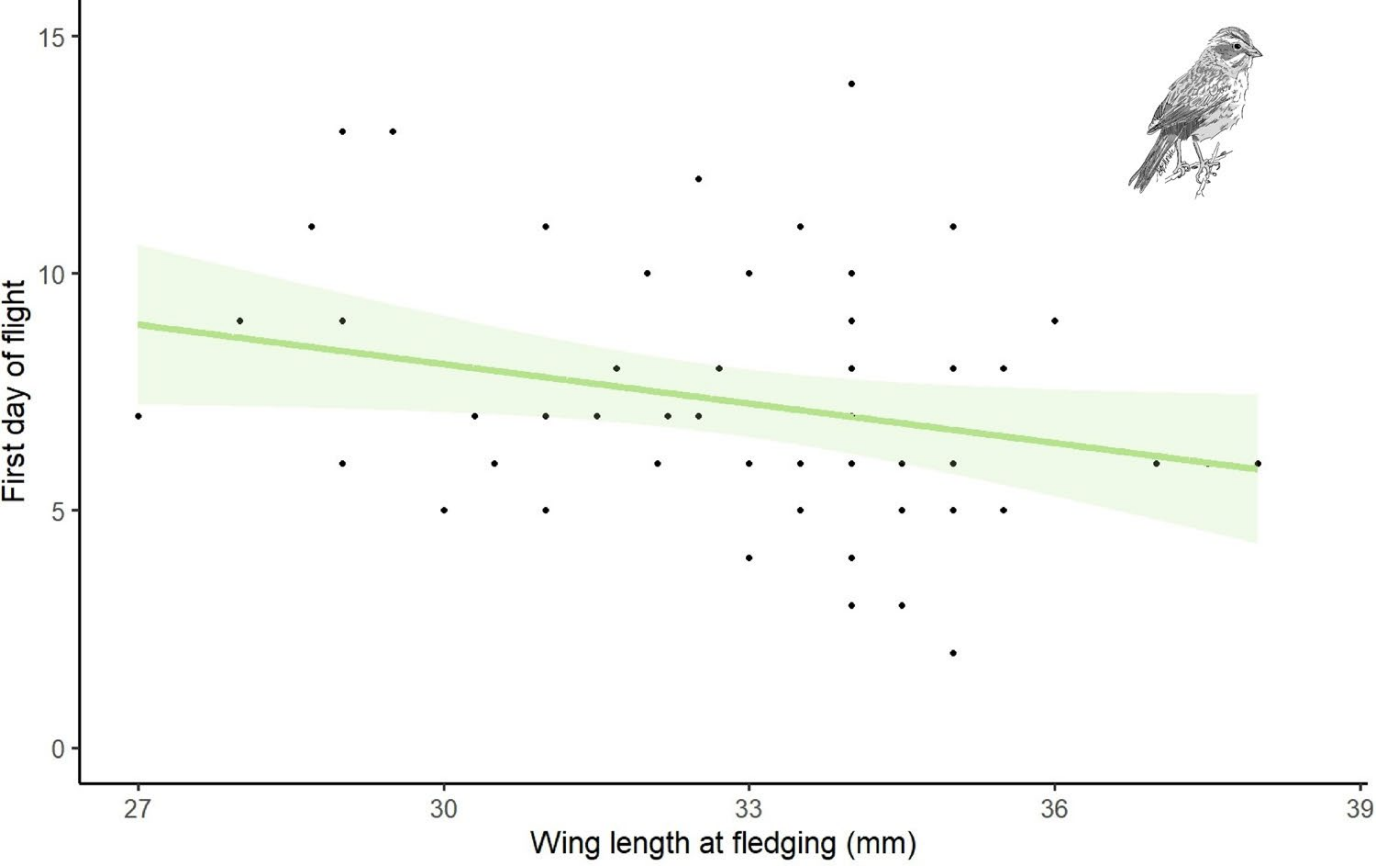
Wing length (mm) at the time of fledging influenced the first day a fledgling was observed flying for Brewer’s sparrows (*n*=60) tracked in the summers of 2021–2023 in Wyoming, USA (*β* = **− **0.28 ± 0.14 SE, *P* = 0.041). Only birds that were observed flying at least once were included in the analysis. The prediction from a GLMM with 95% CI is shown

Brewer’s sparrows with longer tarsi had a reduced risk of overall mortality (a 32% reduction in risk for every 1 mm increase in tarsus length) during the post-fledging period. We found no evidence of a relationship between overall risk of mortality and any of the other traits we considered (Table 2, Fig 3, Online Resource Table S1). Nonetheless, Brewer’s sparrows with longer wings at fledging initiated flight earlier than those with shorter wings (*n* = 60, *β* = − 0.28 ± 0.14 SE, *P* = 0.041) (Fig 4). Wing length was significantly associated with overall mortality risk in sagebrush sparrows (Online Resource Table S2). Sagebrush sparrow nestlings with 1 mm longer wings had a 13% lower risk of post-fledging mortality (Table 2, Fig 3). Both tarsus length and body condition influenced post-fledging mortality for sage thrashers (Online Resource Table S3). Sage thrasher nestlings with 1 mm longer tarsi and higher body condition scores had a 32% and 34% reduced risk of mortality after fledgling, respectively (Table 2, Fig 3).

**Table 2:** Predictor variables influenced overall mortality risk for Brewer’s sparrows (*n*=639 observations, 52 events), sagebrush sparrows (*n*=463 observations, 22 events), and sage thrashers (*n*=414 observations, 21 events) tracked in Wyoming, USA during 2021–2023

Predictor	Relative hazard (*β*)	SE	Hazard ratio (HR)	*P-value*
Brewer’s sparrow				
*Mortality risk ~ tarsus + feather + body condition + (1|Year/nestid)*				
Tarsus	**− 0.30**	**0.10**	**0.74**	0.003*
Feather	**− **0.23	0.16	0.79	0.15
Body condition	**− **0.12	0.15	0.88	0.40
Sagebrush sparrow				
*Mortality risk ~ wing + (1|Year/nestid)* *Mortality risk ~ wing + tarsus + (1|Year/nestid)* *Mortality risk ~ feather + tarsus + (1|Year/nestid)* *Mortality risk ~ wing + feather + (1|Year/NestId)*				
Wing	**− 0.42**	**0.18**	**0.66**	0.017*
Tarsus	**− **0.26	0.22	0.77	0.24
Feather	**− **0.26	0.27	0.77	0.34
Sage thrasher				
*Mortality risk ~ tarsus + body condition + (1|Year/nestid)*				
Tarsus	**− 0.52**	**0.13**	**0.60**	<0.001*
Body condition	**− 0.41**	**0.19**	**0.66**	0.027*

Coefficients were derived from the top mixed-effects Cox model for Brewer’s sparrows and sage thrashers and from the conditional average of all mixed-effects Cox models within ∆ AICc < 2 for sagebrush sparrows. Full AICc model suites are included in the Electronic Supplemental Material (Online Resource Tables S1-S3).

Brewer’s sparrows and sagebrush sparrows with longer tarsi on the day of fledging had a lower risk (43% and 52% reduced risk for each 1 mm increase in length, respectively) of predation post-fledging, whereas no other traits significantly influenced predation risk (Table 3, Fig 3, Online Resource Table S4, Online Resource Table S5). Conversely, the nestling trait that most influenced predation risk for sage thrashers was body condition (Online Resource Table S6). Sage thrasher fledglings with higher body condition scores had a 44% lower risk of predation (Table 3, Fig 3).

**Table 3:** Predictor variables influenced predation risk for Brewer’s sparrows (*n*=598 observations, 33 events), sagebrush sparrows (*n*=448 observations, 13 events), and sage thrashers (*n*=399 observations, 14 events) tracked in Wyoming, USA during 2021-2023

Predictor	Relative hazard (*β*)	SE	Hazard ratio (HR)	*P-value*
Brewer’s sparrow				
*Predation risk ~ tarsus + feather + body condition + (1|Year/nestid)*				
Tarsus	**− 0.45**	**0.16**	**0.64**	0.0054*
Feather	**− **0.002	0.20	1.00	0.99
Body condition	**− **0.19	0.18	0.83	0.31
Sagebrush sparrow				
*Predation risk ~ tarsus + (1|Year/nestid)* *Predation risk ~ feather + tarsus + (1|Year/nestid)* *Predation risk ~ wing + tarsus + (1|Year/nestid)* *Predation risk ~ tarsus + body condition + (1|Year/nestid)*				
Tarsus	**− 0.62**	**0.26**	**0.54**	0.017*
Feather	**− **0.32	0.27	0.73	0.24
Wing	**− **0.20	0.26	0.82	0.45
Body condition	**− **0.07	0.28	0.93	0.80
Sage thrasher				
*Predation risk ~ body condition + (1|Year/nestid)* *Predation risk ~ body condition + tarsus + (1|Year/nestid)* *Predation risk ~ body condition + wing + (1|Year/nestid)* *Predation risk ~ body condition + feather + (1|Year/nestid)*				
Body condition	**− 0.58**	**0.26**	**0.56**	0.022*
Tarsus	**− **0.25	0.24	0.78	0.29
Wing	**− **0.24	0.24	0.79	0.33
Feather	0.12	0.27	1.13	0.64

Coefficients for predation risk were derived from the top mixed-effects Cox model for Brewer’s sparrows and the conditional averages of mixed-effects Cox models within ∆ AICc < 2 for sagebrush sparrows and sage thrashers. Full AICc model suites are included in the Electronic Supplemental Material (Online Resource Tables S4-S6).

The nestling trait most associated with exposure risk in Brewer’s sparrows was feather development at fledging (Online Resource Table S7). Fledglings with more developed feathers had a lower risk of mortality from exposure (Table 4, Fig 3). The minimum temperatures experienced by Brewer’s sparrow fledglings between relocations were also lower for fledglings that died of exposure (*n* = 17, 3.5 °C ± 2.3 SD) than for those that survived (*n* = 564, 6.0 °C ± 2.3 °C SD) (Online Resource Fig S5), corroborating the assumption that exposure-caused mortalities occurred during colder weather. We were unable to test carryover effects on exposure risk for sagebrush sparrows and sage thrashers because of insufficient sample size (*n*=7 exposure mortalities for each species).

**Table 4:** Predictor variables influenced exposure risk for Brewer’s sparrows (*n*=556 observations, 13 events) tracked in Wyoming, USA during 2021-2023

Predictor	Relative hazard (*β*)	SE	Hazard ratio (HR)	*P-value*
Brewer’s sparrow				
*Exposure risk ~ feather + (1|Year/nestid)* *Exposure risk ~ feather + tarsus + (1|Year/nestid)* *Exposure risk ~ feather + body condition + (1|Year/nestid)*				
Feather	**− 1.16**	**0.34**	**0.31**	<0.001*
Tarsus	**− **0.14	0.14	0.87	0.30
Wing	0.12	0.29	1.13	0.68

Coefficients for exposure risk in Brewer’s sparrows were derived from conditional averages of mixed-effects Cox models within ∆ AICc < 2. Full AICc model suites are included in the Electronic Supplemental Material (Online Resource Table S7).

## Discussion

Morphological development during early life-stages can carry over to affect survival in later stages, by influencing mobility or body condition and therefore the ability of young animals to respond to risks (Jones and Ward 2020). The traits that mostly influence survival, however, likely differ depending on the levels and types of risks that animals face in later stages (Jones et al. 2017). Mortality during the post-fledging period was relatively high for Brewer’s sparrows, sagebrush sparrows, and sage thrashers compared with the earlier nestling period, and post-fledging period risks may therefore exert selection pressure on nestling traits (Quinn and Kinnison 1999; Chaparro-Pedraza 2024). Consistent with our *Cause-specific carryover hypothesis*, the morphology of nestlings was carried over to influence post-fledging mortality, and the relative influence of morphological traits varied by risk type. In contrast to prior studies of post-fledging survival in passerines (e.g., Jones et al. 2017; Martin et al. 2018; Vitz and Rodewald 2011), tarsus rather than wing length most influenced the risk of predation in Brewer’s sparrows and sagebrush sparrows, highlighting a previously underappreciated mode of fleeing from predators. By contrast, feather development played a key role in reducing the risk of weather-related mortality for Brewer’s sparrow fledglings. The manifestation of carryover effects from early development therefore appears to be under selection and dependent upon primary causes of mortality.

The overall risk of juvenile mortality and the role of carryover effects across juvenile periods differed between co-occurring species, even though all three experienced similar extrinsic conditions (habitat, predator assemblage, and weather) during the post-fledging period. Sage thrashers had the lowest overall risk of post-fledging mortality and predation, whereas Brewer’s sparrows and sagebrush sparrows experienced higher mortality rates. Tarsus length on the day of fledging most affected overall mortality in Brewer’s sparrows and sage thrashers, whereas wing length had the strongest influence on mortality in sagebrush sparrows. Cause-specific carryover effects varied as well; tarsus length influenced predation risk for both sparrow species, whereas body condition influenced predation risk for sage thrashers. Variation in mortality risk may have resulted from differences in the behavioral, morphological, or life-history traits of the three species. Sage thrashers—the largest and most developed of the three species at fledging age—were more mobile and therefore likely better able to evade predators compared with Brewer’s sparrows or sagebrush sparrows. Post-fledging predation pressure also may have depended on body size (Sinclair et al. 2003). Nest predator assemblages within the same study system differed between Brewer’s sparrows and sage thrashers, potentially related to body size (Hethcoat and Chalfoun 2015b, Chalfoun unpublished data). These differences in carryover effects on post-fledging survival across three sympatric species highlight the importance of recognizing how risk during sensitive periods can vary across related species even within the same habitat.

We consistently observed that fledglings with larger or more developed morphological traits (e.g., longer tarsi) had a decreased risk of mortality. We therefore tested model suites with a body size index to assess the possibility that larger nestling traits could simply reflect larger overall nestling size. The addition of body size, however, did not influence results. Tarsus length often is used as a proxy for body size in avian studies (Freeman and Jackson 1990; Senar and Pascual 1997). However, our findings indicate that different morphological traits including the length of tarsi, wings, and feathers had additive effects on mortality independent of body size.

Assessing carryover effects without explicit consideration of specific causes of mortality can obscure underlying mechanisms, because different traits may be linked to avoidance of different risks. Predation was the most common cause of post-fledging mortality for all three species, and tarsus length at fledging influenced predation risk in Brewer’s sparrows and sagebrush sparrows. Researchers historically have used wing length as a proxy for juvenile mobility or dispersal ability in avian studies (e.g., Claramunt 2021; Sheard et al. 2020; Weeks et al. 2022). However, we observed that the period of greatest mortality in our system occurred during the first 5 days after fledging, when fledglings were not yet capable of flight. The ability of fledglings to run or navigate through dense vegetation when escaping predators therefore likely had a greater influence on mortality risk than wing development (Heers 2023). Running ability may be particularly important in this system because open-cup nesting and shrub-nesting species have more altricial life-history strategies with shortened nesting periods compared with cavity-nesting or forest-dwelling species (Şahin Arslan and Martin 2022). Future studies, particularly in altricial species, may benefit by considering not only species-specific but also stage-specific behavior when assessing which morphological traits may have the greatest influence on mortality during particularly risky life-stages.

### Trade-offs in trait investment during development

Juvenile growth is constrained by both time and resources, which can lead to trade-offs in trait investment (Stearns 1989; Starck and Ricklefs 1998; Stevens et al. 2000). The underappreciated effect of tarsus length on survival during the brief but risky period before fledglings were capable of flight suggests that developing nestlings must trade-off allocation of energy into certain traits during development. For example, investment in longer tarsi could improve survival during the earliest, and riskiest, part of the post-fledging period by increasing mobility pre-flight, whereas investment in longer wings could influence survival during the later post-fledging period and beyond (e.g., dispersal and migration; Chu and Claramunt 2023). Indeed, we observed fledgling Brewer’s sparrows with longer wings flying at a younger age compared with those with shorter wings, indicating that investment in wing length may reduce the duration of the risky flightless period. We were unable to detect carryover effects on the late post-fledging period because of low rates of mortality and high rates of dispersal. Nonetheless, carryover effects through the later juvenile period, including dispersal and migration, remain likely (Evans et al. 2020; Hayes et al. 2024). Animals may invest in certain traits, such as longer tarsi, that influence survival during key periods of high risk, potentially at the expense of traits that influence performance during other, less risky periods (Schluter et al. 1991; Moore and Martin 2019).

Carryover effects differed between Brewer’s sparrow fledglings that died of exposure compared with those that died of predation. Specifically, the extent of feather development rather than tarsus length decreased the risk of exposure-related mortality, likely because feathers provide a thermoregulatory function (Terrill and Shultz 2023). Nestlings therefore may also face a trade-off between investment in traits that influence different types of risk during the post-fledging period. Indeed, within the same study area, weather patterns during nestling development influenced investment in specific traits; nestlings developing in colder conditions invested in higher body condition whereas those developing in warmer conditions grew longer wings (Sanders and Chalfoun in preparation). Notably, anthropogenic changes may alter the kinds of risks that juveniles face, whether through altered predator assemblages (Smith and Dwyer 2016; Sanders and Chalfoun 2019) or shifting weather patterns and associated exposure (Calvin et al. 2023). If risks become increasingly unpredictable or stochastic, mismatches between investments in particular traits during development and the subsequent fitness outcomes may become more prevalent.

## Conclusions

Selective pressures often differ across life-stages (Pearl and Miner 1935; Medina et al. 2019), and therefore, the relative influence of traits can vary depending on stage-specific risks and behavior. We found that the post-fledging period was characterized by high levels of risk for three arid-land songbirds. Furthermore, carryover effects of nestling morphology on survival during the post-fledging period varied depending on the cause of mortality (*Cause-specific carryover hypothesis*), thus highlighting a potential trade-off in trait investment during nestling development. When selective processes associated with specific life-stages are altered by anthropogenic change, disentangling the factors underlying survival during those sensitive life-stages may be vital for conservation. We suggest that the traits that most influence survival are shaped by stage- and cause-specific levels of risk, as well as the behaviors that animals exhibit during limiting periods.

## Supplementary Information

Below is the link to the electronic supplementary material.

Supplementary file1 (DOCX 4100 KB)

## Data Availability

Data are available at Shertzer, E.R., Jones, D.W., and Chalfoun, A.D., 2025, Radio telemetry tracking data for sagebrush songbird fledglings in Wyoming, between 2021-2023: U.S. Geological Survey data release, 10.5066/P14KCRME.

## Declarations

### Highlighted student paper

Carryover effects on cause-specific mortality are rarely investigated, particularly for juvenile periods. We demonstrate how physiology and behavior during risky stages create investment trade-offs.

### Code availability

Code is available at https://github.com/eshertzer/Shertzeretal2025_Oecologia.
