# Supplementary material for: Carryover effects across juvenile periods vary by species, cause-specific mortality, and stage-specific behavior
Source: Oecologia. 2026 Jun 19;208(7):82. doi: 10.1007/s00442-026-05887-5 (PMC13282251; doi:10.1007/s00442-026-05887-5)
Supplement: Supplementary file 1 — Supplementary file1 (DOCX 4100 KB) [file 442_2026_5887_MOESM1_ESM.docx]

**Electronic Supplemental Material**

**Title:** Carryover effects across juvenile periods vary by species, cause-specific mortality, and stage-specific behavior

**Authors**: Emily R. Shertzer (Corresponding author: [eshertze@uwyo.edu](mailto:eshertze@uwyo.edu), 1-610-308-7564)^1^, Donald W. Jones^1^, Anna D. Chalfoun^1, 2^

**Affiliations:** 1. Wyoming Cooperative Fish and Wildlife Research Unit, Department of Zoology and Physiology and Program in Ecology and Evolution, University of Wyoming, Laramie WY USA; 2. United States Geological Survey.


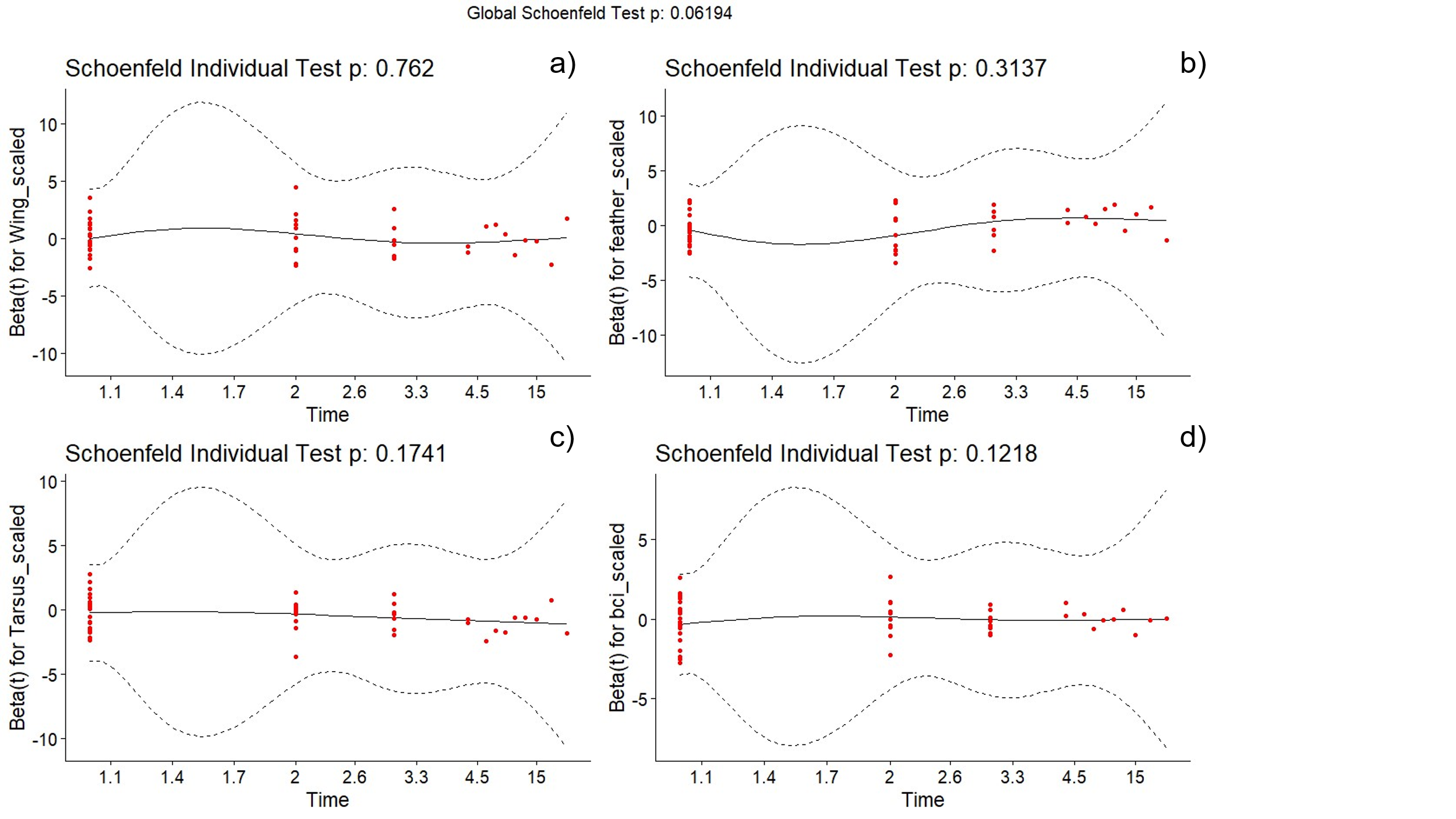
**Fig. S1** Schoenfeld residual tests of wing length (a), feather emergence (b), tarsus length (c), and body condition (d) in Brewer’s sparrows were not significant, indicating that the assumption of the Cox proportional hazards model, that the effects of hazards did not change over the study period, was not violated.


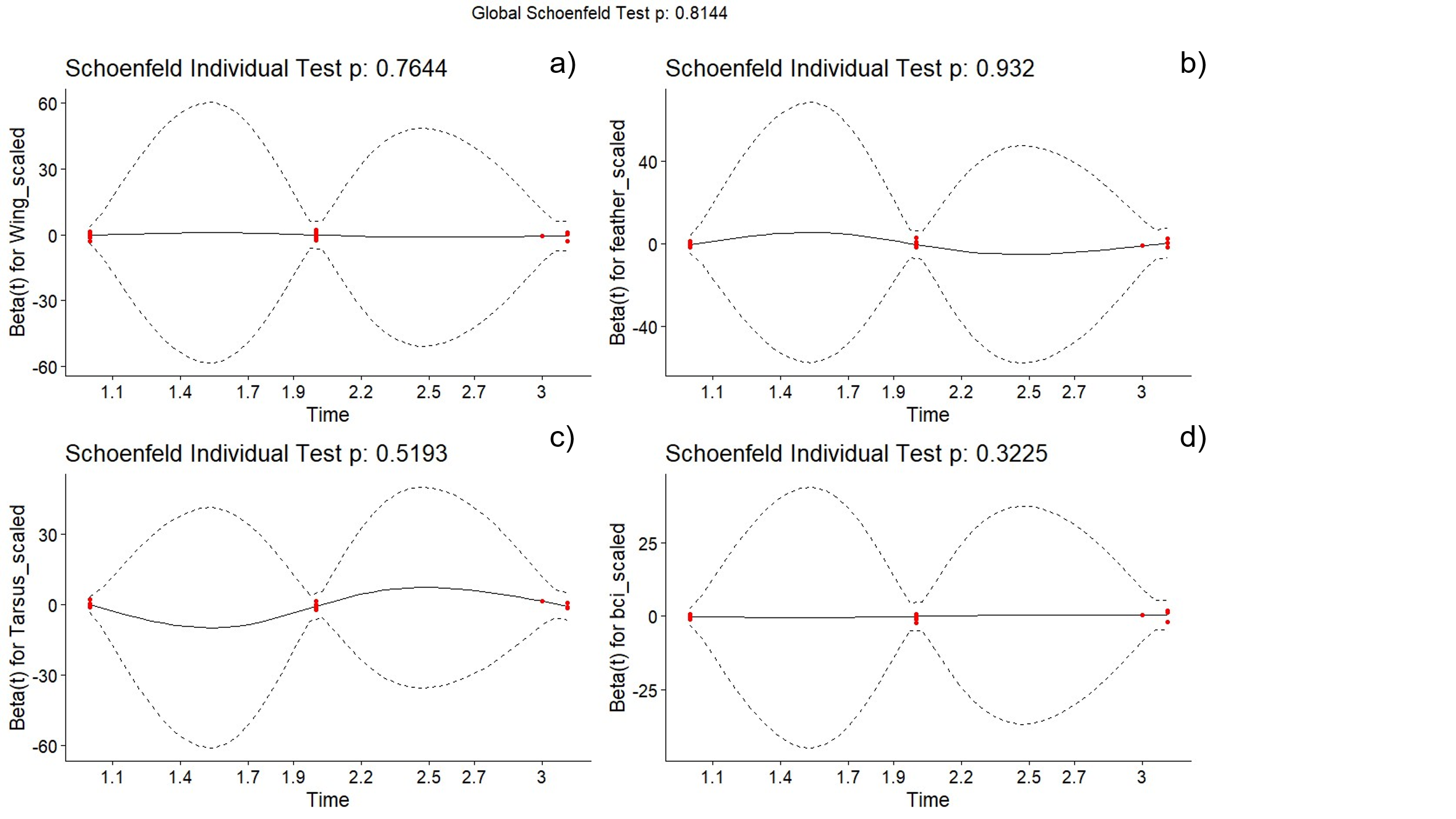
**Fig. S2** Schoenfeld residual tests of wing length (a), feather emergence (b), tarsus length (c), and body condition (d) in sagebrush sparrows were not significant, indicating that the assumption of the Cox proportional hazards model, that the effects of hazards did not change over the study period, was not violated.


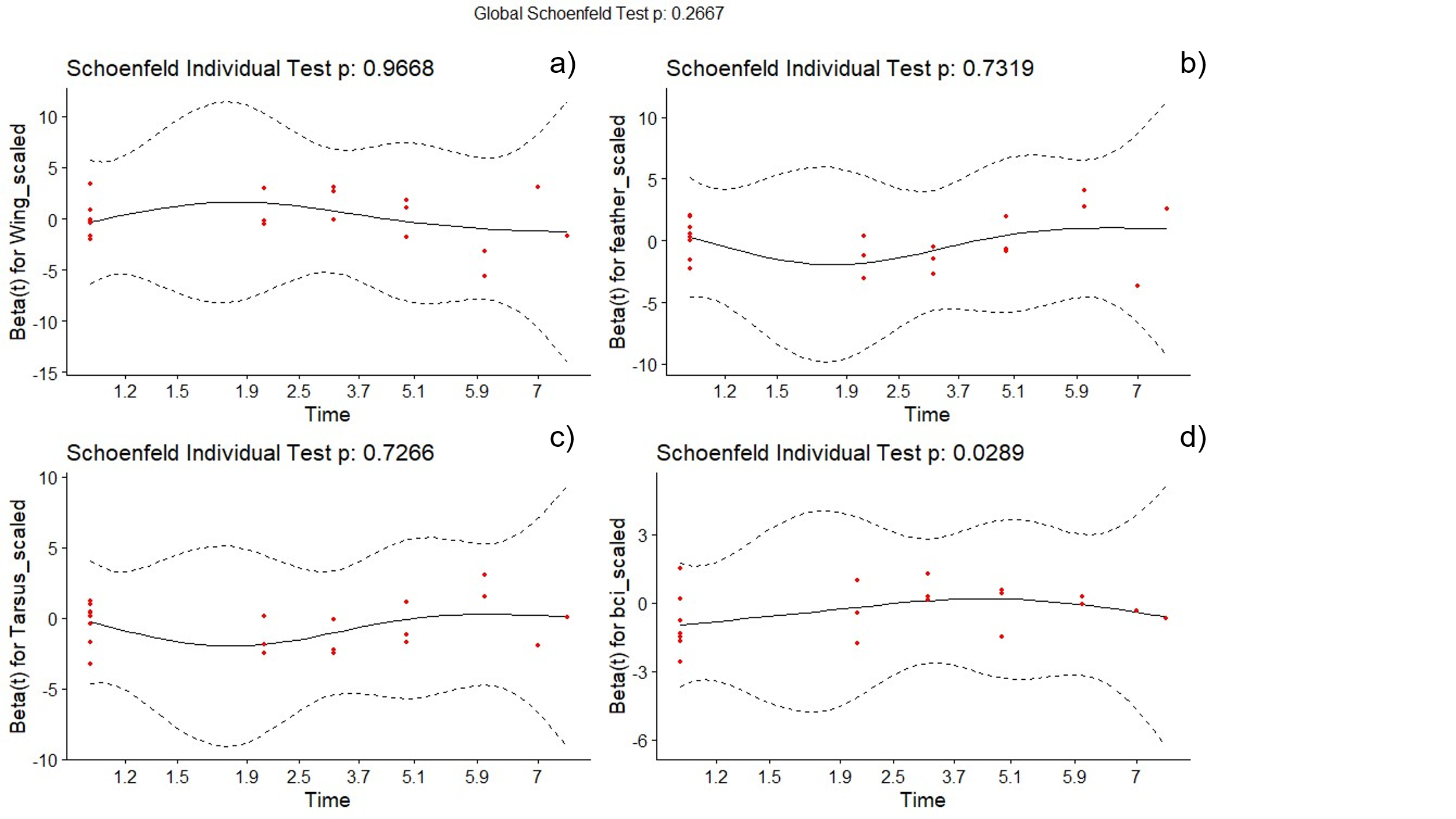


**Fig. S3** Schoenfeld residual tests of wing length (a), feather emergence (b), and tarsus length (c) in sage thrashers were not significant, indicating that the assumption of the Cox proportional hazards model, that the effects of hazards did not change over the study period, was not violated. The Schoenfeld residual test was significant for body condition (d). However, we determined that the significant result for body condition index would not affect the interpretation of our results and may in fact have been driven by low sample sizes after the first day of the post-fledging period.


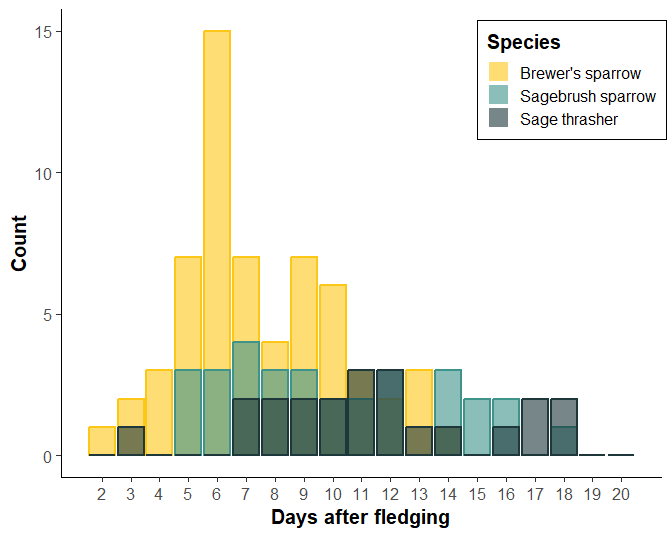
**Fig. S4** Flight was typically first observed around the fifth through seventh day after fledging in Brewer’s sparrows, sagebrush sparrows, and sage thrashers tracked during the summers of 2021-2023 in Wyoming, USA. Flight initiation was recorded on the first day an individual was observed flying, but the first observed day of flight was not necessarily the first day an individual was capable of flight.

|  | K | AICc | ∆ AICc | ModelLik | AICcWt | LL | Cum.Wt |
| --- | --- | --- | --- | --- | --- | --- | --- |
| **Feather + Tarsus + BCI + (1\|Year/NestId)** | **5** | **434.30** | **0.00** | **1.00** | **0.90** | **-212.10** | **0.91** |
| Feather + BCI + (1\|Year/NestId) | 4 | 439.10 | 4.80 | 0.09 | 0.08 | -215.52 | 0.99 |
| Feather + Tarsus + (1\|Year/NestId) | 4 | 443.35 | 9.04 | 0.01 | 0.01 | -217.64 | 1.00 |
| Feather + (1\|Year/NestId) | 3 | 447.94 | 13.64 | 0.00 | 0.00 | -220.95 | 1.00 |
| Tarsus + BCI + (1\|Year/NestId) | 4 | 513.39 | 79.09 | 0.00 | 0.00 | -252.67 | 1.00 |
| Wing + Tarsus + BCI + (1\|Year/NestId) | 5 | 514.52 | 80.22 | 0.00 | 0.00 | -252.22 | 1.00 |
| Wing + BCI + (1\|Year/NestId) | 4 | 515.57 | 81.27 | 0.00 | 0.00 | -253.76 | 1.00 |
| BCI + (1\|Year/NestId) | 3 | 530.55 | 96.25 | 0.00 | 0.00 | -262.26 | 1.00 |
| Wing + Tarsus + (1\|Year/NestId) | 4 | 534.69 | 100.39 | 0.00 | 0.00 | -263.31 | 1.00 |
| Wing + (1\|Year/NestId) | 3 | 534.83 | 100.53 | 0.00 | 0.00 | -264.40 | 1.00 |
| Tarsus + (1\|Year/NestId) | 3 | 535.55 | 101.25 | 0.00 | 0.00 | -264.76 | 1.00 |
| Null | 2 | 556.12 | 121.82 | 0.00 | 0.00 | -276.05 | 1.00 |

**Table S1** Model selection table for mixed-effects Cox proportional hazards models to analyse mortality risk in Brewer’s sparrow fledglings tracked in Wyoming, USA during 2021-2023. The top model was selected with ∆ AICc > 2 as compared to other models within the suite. BCI refers to the body condition index.

**Table S2** Model selection table for mixed-effects Cox proportional hazards models to analyse mortality risk in sagebrush sparrow fledglings tracked in Wyoming, USA during 2022-2023. All models within ∆AICc < 2 were model averaged. BCI refers to the body condition index.

|  | K | AICc | ∆ AICc | ModelLik | AICcWt | LL | Cum.Wt |
| --- | --- | --- | --- | --- | --- | --- | --- |
| **Wing + (1\|Year/NestId)** | **3** | **166.127** | **0.000** | **1.000** | **0.238** | **-80.037** | **0.238** |
| **Wing + Tarsus + (1\|Year/NestId)** | **4** | **167.453** | **1.326** | **0.515** | **0.122** | **-79.683** | **0.360** |
| **Feather + Tarsus + (1\|Year/NestId)** | **4** | **167.626** | **1.499** | **0.473** | **0.112** | **-79.769** | **0.472** |
| **Wing + Feather + (1\|Year/NestId)** | **4** | **167.896** | **1.769** | **0.413** | **0.098** | **-79.905** | **0.570** |
| Wing + BCI + (1\|Year/NestId) | 4 | 168.145 | 2.018 | 0.365 | 0.087 | -80.029 | 0.657 |
| Tarsus + (1\|Year/NestId) | 3 | 168.689 | 2.562 | 0.278 | 0.066 | -81.319 | 0.723 |
| Feather + (1\|Year/NestId) | 3 | 168.938 | 2.811 | 0.245 | 0.058 | -81.443 | 0.781 |
| Wing + Feather + Tarsus + (1\|Year/NestId) | 5 | 169.008 | 2.881 | 0.237 | 0.056 | -79.438 | 0.837 |
| Wing + Tarsus + BCI + (1\|Year/NestId) | 5 | 169.490 | 3.363 | 0.186 | 0.044 | -79.679 | 0.882 |
| Feather + Tarsus + BCI + (1\|Year/NestId) | 5 | 169.602 | 3.475 | 0.176 | 0.042 | -79.736 | 0.923 |
| Tarsus + BCI + (1\|Year/NestId) | 4 | 170.661 | 4.534 | 0.104 | 0.025 | -81.287 | 0.948 |
| Feather + BCI + (1\|Year/NestId) | 4 | 170.919 | 4.792 | 0.091 | 0.022 | -81.416 | 0.970 |
| Wing + Feather + Tarsus + BCI + (1\|Year/NestId) | 6 | 171.026 | 4.899 | 0.086 | 0.021 | -79.421 | 0.990 |
| Null | 2 | 173.133 | 7.006 | 0.030 | 0.007 | -84.554 | 0.997 |
| BCI + (1\|Year/NestId) | 3 | 175.122 | 8.995 | 0.011 | 0.003 | -84.535 | 1.000 |
|  |  |  |  |  |  |  |  |

**Table S3** Model selection table for mixed-effects Cox proportional hazards models to analyse mortality risk in sage thrasher fledglings tracked in Wyoming, USA during 2022-2023. The top model was selected with ∆ AICc > 2 as compared to all other models within the model suite. BCI refers to the body condition index.

|  | K | AICc | ∆ AICc | ModelLik | AICcWt | LL | Cum.Wt |
| --- | --- | --- | --- | --- | --- | --- | --- |
| **Tarsus + BCI + (1\|Year/NestId)** | **4** | **156.134** | **0** | **1** | **0.359** | **-74.018** | **0.359** |
| Wing + Tarsus + BCI + (1\|Year/NestId) | 5 | 158.167 | 2.033 | 0.361 | 0.130 | -74.010 | 0.490 |
| Feather + Tarsus + BCI + (1\|Year/NestId) | 5 | 158.172 | 2.0380 | 0.361 | 0.130 | -74.012 | 0.619 |
| Tarsus + (1\|Year/NestId) | 3 | 158.626 | 2.492 | 0.288 | 0.103 | -76.284 | 0.723 |
| Wing + BCI + (1\|Year/NestId) | 4 | 158.801 | 2.667 | 0.263 | 0.095 | -75.352 | 0.817 |
| Wing + Feather + Tarsus + BCI + (1\|Year/NestId) | 6 | 160.225 | 4.092 | 0.129 | 0.046 | -74.009 | 0.864 |
| Wing + Tarsus + (1\|Year/NestId) | 4 | 160.661 | 4.527 | 0.104 | 0.037 | -76.282 | 0.901 |
| Feather + Tarsus + (1\|Year/NestId) | 4 | 160.664 | 4.530 | 0.104 | 0.037 | -76.283 | 0.939 |
| Wing + (1\|Year/NestId) | 3 | 161.214 | 5.081 | 0.079 | 0.028 | -77.578 | 0.967 |
| Wing + Feather + Tarsus + (1\|Year/NestId) | 5 | 162.701 | 6.568 | 0.037 | 0.013 | -76.277 | 0.980 |
| Wing + Feather + (1\|Year/NestId) | 4 | 162.848 | 6.715 | 0.035 | 0.012 | -77.375 | 0.993 |
| BCI + (1\|Year/NestId) | 3 | 165.681 | 9.548 | 0.008 | 0.003 | -79.812 | 0.996 |
| Feather + BCI + (1\|Year/NestId) | 4 | 165.683 | 9.550 | 0.008 | 0.003 | -78.793 | 0.999 |
| Feather + (1\|Year/NestId) | 3 | 169.071 | 12.937 | 0.002 | 0.001 | -81.506 | 1.000 |
| Null | 2 | 169.356 | 13.223 | 0.001 | 0.000 | -82.663 | 1.000 |

**Table S4** Model selection table for mixed-effects Cox proportional hazards models to analyse predation risk in Brewer’s sparrow fledglings tracked in Wyoming, USA during 2021-2023. The top model was selected with ∆ AICc > 2 as compared to other models within the suite. BCI refers to the body condition index.

|  | K | AICc | ∆ AICc | ModelLik | AICcWt | LL | Cum.Wt |
| --- | --- | --- | --- | --- | --- | --- | --- |
| **Feather + Tarsus + BCI + (1\|Year/NestId)** | **5** | **271.23** | **0.00** | **1.00** | **0.90** | **-130.57** | **0.90** |
| Feather + BCI + (1\|Year/NestId) | 4 | 275.96 | 4.73 | 0.09 | 0.09 | -133.95 | 0.99 |
| Feather + Tarsus + (1\|Year/NestId) | 4 | 280.29 | 9.06 | 0.01 | 0.01 | -136.11 | 1.00 |
| Feather + (1\|Year/NestId) | 3 | 285.38 | 14.15 | 0.00 | 0.00 | -139.67 | 1.00 |
| Tarsus + BCI + (1\|Year/NestId) | 4 | 299.22 | 27.98 | 0.00 | 0.00 | -145.58 | 1.00 |
| Wing + Tarsus + BCI + (1\|Year/NestId) | 5 | 301.25 | 30.02 | 0.00 | 0.00 | -145.58 | 1.00 |
| Wing + BCI + (1\|Year/NestId) | 4 | 303.56 | 32.33 | 0.00 | 0.00 | -147.75 | 1.00 |
| BCI + (1\|Year/NestId) | 3 | 308.67 | 37.43 | 0.00 | 0.00 | -151.31 | 1.00 |
| Tarsus + (1\|Year/NestId) | 3 | 317.87 | 46.64 | 0.00 | 0.00 | -155.92 | 1.00 |
| Wing + Tarsus+ (1\|Year/NestId) | 4 | 319.32 | 48.09 | 0.00 | 0.00 | -155.63 | 1.00 |
| Wing + (1\|Year/NestId) | 3 | 320.60 | 49.37 | 0.00 | 0.00 | -157.28 | 1.00 |
| Null | 2 | 330.66 | 59.43 | 0.00 | 0.00 | -163.32 | 1.00 |

**Table S5** Model selection table for mixed-effects Cox proportional hazards models to analyse predation risk in sagebrush sparrow fledglings tracked in Wyoming, USA during 2022-2023. All models within ∆ AICc < 2 were model averaged. BCI refers to the body condition index.

|  | K | AICc | ∆ AICc | ModelLik | AICcWt | LL | Cum.Wt |
| --- | --- | --- | --- | --- | --- | --- | --- |
| **Tarsus + (1\|Year/NestId)** | **3** | **94.551** | **0** | **1** | **0.229** | **-44.249** | **0.229** |
| **Feather + Tarsus + (1\|Year/NestId)** | **4** | **95.104** | **0.553** | **0.759** | **0.174** | **-43.507** | **0.402** |
| **Wing + Tarsus + (1\|Year/NestId)** | **4** | **96.043** | **1.491** | **0.474** | **0.109** | **-43.976** | **0.511** |
| **Tarsus + BCI + (1\|Year/NestId)** | **4** | **96.521** | **1.970** | **0.373** | **0.085** | **-44.216** | **0.597** |
| Wing + (1\|Year/NestId) | 3 | 96.665 | 2.114 | 0.347 | 0.080 | -45.306 | 0.676 |
| Feather + Tarsus + BCI + (1\|Year/NestId) | 5 | 97.072 | 2.521 | 0.284 | 0.065 | -43.468 | 0.741 |
| Wing + Feather + Tarsus + (1\|Year/NestId) | 5 | 97.103 | 2.552 | 0.279 | 0.064 | -43.484 | 0.805 |
| Feather + (1\|Year/NestId) | 3 | 97.939 | 3.387 | 0.184 | 0.042 | -45.942 | 0.847 |
| Wing + Tarsus + BCI + (1\|Year/NestId) | 5 | 98.061 | 3.510 | 0.173 | 0.040 | -43.963 | 0.887 |
| Wing + Feather + (1\|Year/NestId) | 4 | 98.446 | 3.894 | 0.143 | 0.033 | -45.178 | 0.919 |
| Wing + BCI + (1\|Year/NestId) | 4 | 98.698 | 4.147 | 0.126 | 0.029 | -45.304 | 0.948 |
| Wing + Feather + Tarsus + BCI + (1\|Year/NestId) | 6 | 99.065 | 4.513 | 0.105 | 0.024 | -43.437 | 0.972 |
| Feather + BCI + (1\|Year/NestId) | 4 | 99.969 | 5.418 | 0.067 | 0.015 | -45.939 | 0.987 |
| Null | 2 | 100.946 | 6.394 | 0.041 | 0.009 | -48.459 | 0.997 |
| BCI + (1\|Year/NestId) | 3 | 102.97 | 8.421 | 0.015 | 0.003 | -48.459 | 1 |

**Table S6** Model selection table for mixed-effects Cox proportional hazards models to analyse predation risk in sage thrasher fledglings tracked in Wyoming, USA during 2022-2023. Models within ∆ AICc < 2 were model averaged. BCI refers to the body condition index.

|  | K | AICc | ∆ AICc | ModelLik | AICcWt | LL | Cum.Wt |
| --- | --- | --- | --- | --- | --- | --- | --- |
| **BCI + (1\|Year/NestId)** | **3** | **109.225** | **0.000** | **1.000** | **0.249** | **-51.582** | **0.249** |
| **Tarsus + BCI + (1\|Year/NestId)** | **4** | **110.229** | **1.004** | **0.605** | **0.151** | **-51.064** | **0.399** |
| **Wing + BCI + (1\|Year/NestId)** | **4** | **110.357** | **1.132** | **0.568** | **0.141** | **-51.128** | **0.541** |
| **Feather + BCI + (1\|Year/NestId)** | **4** | **111.046** | **1.821** | **0.402** | **0.100** | **-51.472** | **0.641** |
| Feather + Tarsus + BCI + (1\|Year/NestId) | 5 | 111.824 | 2.599 | 0.273 | 0.068 | -50.836 | 0.709 |
| Wing + Tarsus + BCI + (1\|Year/NestId) | 5 | 112.116 | 2.892 | 0.236 | 0.059 | -50.982 | 0.767 |
| Wing + Feather + Tarsus + BCI + (1\|Year/NestId) | 6 | 112.463 | 3.238 | 0.198 | 0.049 | -50.125 | 0.817 |
| Tarsus + (1\|Year/NestId) | 3 | 112.738 | 3.513 | 0.173 | 0.043 | -53.339 | 0.860 |
| Null | 2 | 112.879 | 3.655 | 0.161 | 0.040 | -54.425 | 0.900 |
| Feather + Tarsus + (1\|Year/NestId) | 4 | 114.003 | 4.778 | 0.092 | 0.023 | -52.951 | 0.922 |
| Wing + (1\|Year/NestId) | 3 | 114.126 | 4.901 | 0.086 | 0.021 | -54.033 | 0.944 |
| Feather + (1\|Year/NestId) | 3 | 114.653 | 5.428 | 0.066 | 0.016 | -54.296 | 0.960 |
| Wing + Tarsus + (1\|Year/NestId) | 4 | 114.745 | 5.521 | 0.063 | 0.016 | -53.322 | 0.976 |
| Wing + Feather + (1\|Year/NestId) | 4 | 114.837 | 5.612 | 0.060 | 0.015 | -53.368 | 0.991 |
| Wing + Feather + Tarsus + (1\|Year/NestId) | 5 | 115.913 | 6.688 | 0.035 | 0.009 | -52.880 | 1.000 |

**Table S7** Model selection table for mixed-effects Cox proportional hazards models to analyse exposure risk in Brewer’s sparrow fledglings tracked in Wyoming, USA during 2021-2023. All models within ∆ AICc < 2 were model averaged. BCI refers to the body condition index.

|  | K | AICc | ∆ AICc | ModelLik | AICcWt | LL | Cum.Wt |
| --- | --- | --- | --- | --- | --- | --- | --- |
| **Feather + (1\|Year/NestId)** | **3** | **91.35** | **0.00** | **1.00** | **0.44** | **-42.65** | **0.44** |
| **Feather + Tarsus + (1\|Year/NestId)** | **4** | **92.37** | **1.02** | **0.60** | **0.27** | **-42.15** | **0.71** |
| **Feather + BCI + (1\|Year/NestId)** | **4** | **93.11** | **1.76** | **0.41** | **0.18** | **-42.52** | **0.90** |
| Feather + Tarsus + BCI + (1\|Year/NestId) | 5 | 94.26 | 2.91 | 0.23 | 0.10 | -42.08 | 1.00 |
| Wing + (1\|Year/NestId) | 3 | 132.42 | 41.07 | 0.00 | 0.00 | -63.19 | 1.00 |
| Wing + BCI + (1\|Year/NestId) | 4 | 132.90 | 41.54 | 0.00 | 0.00 | -62.41 | 1.00 |
| Wing + Tarsus + (1\|Year/NestId) | 4 | 134.33 | 42.98 | 0.00 | 0.00 | -63.13 | 1.00 |
| Wing + Tarsus + BCI + (1\|Year/NestId) | 5 | 134.90 | 43.55 | 0.00 | 0.00 | -62.40 | 1.00 |
| Tarsus + BCI + (1\|Year/NestId) | 4 | 137.03 | 45.67 | 0.00 | 0.00 | -64.48 | 1.00 |
| Tarsus + (1\|Year/NestId) | 3 | 137.74 | 46.39 | 0.00 | 0.00 | -65.85 | 1.00 |
| BCI + (1\|Year/NestId) | 3 | 147.47 | 56.12 | 0.00 | 0.00 | -70.72 | 1.00 |
| Null | 2 | 149.58 | 58.23 | 0.00 | 0.00 | -72.78 | 1.00 |

**Table S8** Model outputs for mixed-effects Cox proportional hazards models to analyse how mortality (n = 1561 observations, 106 events), predation (n = 1487 observations, 65 events), and exposure risk (n= 1402 observations, 31 events) varied between Brewer’s sparrow, sagebrush sparrow, and sage thrasher fledglings tracked in Wyoming, USA during 2021-2023. Brewer’s sparrow is the reference species in model outputs.

| Predictor | Relative hazard (β) | SE | Hazard ratio (HR) | *P-value* |
| --- | --- | --- | --- | --- |
| *mortality risk ~ species + (1\|Year/NestId)* | | | | |
| Sagebrush sparrow | -0.23 | 0.28 | 0.80 | 0.43 |
| **Sage thrasher** | **-0.63** | **0.29** | **0.53** | **0.027*** |
| *predation risk ~ species + (1\|Year/NestId)* | | | | |
| Sagebrush sparrow | -0.39 | 0.34 | 0.68 | 0.26 |
| Sage thrasher | -0.60 | 0.33 | 0.55 | 0.07 |
| *exposure risk ~ species + (1\|Year/NestId)* | | | | |
| Sagebrush sparrow | -0.13 | 0.53 | 0.88 | 0.81 |
| Sage thrasher | -0.69 | 0.53 | 0.50 | 0.20 |

**Table S9** Kaplan-Meier estimates of survival by day for nestlings and fledglings tracked in Wyoming, USA during 2021-2023.

|  | Nestling period | | | | | Fledgling period | | | | |
| --- | --- | --- | --- | --- | --- | --- | --- | --- | --- | --- |
| *Brewer’s sparrow* | | | | | | | | | | |
| Day | n | n(event) | Survival estimate | SE | 95% CI | n | n(event) | Survival estimate | SE | 95% CI |
| 1 | 2526 | 25 | 0.990 | 0.002 | 0.986-0.994 | 128 | 25 | 0.805 | 0.035 | 0.739-0.876 |
| 2 | 2134 | 14 | 0.984 | 0.003 | 0.979-0.989 | 102 | 14 | 0.694 | 0.041 | 0.619-0.779 |
| 3 | 1818 | 20 | 0.973 | 0.004 | 0.966-0.980 | 86 | 11 | 0.605 | 0.044 | 0.526-0.697 |
| 4 | 1498 | 37 | 0.949 | 0.005 | 0.939-0.959 | 75 | 2 | 0.589 | 0.044 | 0.509-0.682 |
| 5 | 1170 | 52 | 0.907 | 0.008 | 0.892-0.922 | 73 | 1 | 0.581 | 0.044 | 0.501-0.674 |
| 6 | 552 | 23 | 0.869 | 0.011 | 0.848-0.890 | *No change* | | | | |
| 7 | 312 | 30 | 0.785 | 0.017 | 0.752-0.820 | 65 | 1 | 0.572 | 0.044 | 0.492-0.666 |
| *Sagebrush sparrow* | | | | | | | | | | |
| 1 | 988 | 2 | 0.998 | 0.001 | 0.995-1.000 | 59 | 9 | 0.847 | 0.047 | 0.761-0.944 |
| 2 | 841 | 8 | 0.988 | 0.004 | 0.981-0.996 | 50 | 9 | 0.695 | 0.060 | 0.587-0.823 |
| 3 | 709 | 6 | 0.980 | 0.005 | 0.970-0.990 | 41 | 1 | 0.678 | 0.061 | 0.569-0.808 |
| 4 | 591 | 11 | 0.962 | 0.007 | 0.948-0.976 | *No change* | | | | |
| 5 | 487 | 7 | 0.948 | 0.009 | 0.931-0.966 |  |  |  |  |  |
| 6 | 380 | 15 | 0.911 | 0.013 | 0.886-0.936 | 38 | 3 | 0.624 | 0.063 | 0.512-0.762 |
| 7 | 196 | 3 | 0.897 | 0.015 | 0.868-0.926 | *No change* | | | | |
| 8 | 90 | 1 | 0.887 | 0.018 | 0.853-0.922 |  |  |  |  |  |
| *Sage thrasher* | | | | | | | | | | |
| 1 |  |  |  |  | 0.989-0.000 | 68 | 8 | 0.882 | 0.039 | 0.809-0.962 |
| 2 | 876 | 5 | 0.994 | 0.003 | 0.989-0.000 | 56 | 3 | 0.835 | 0.046 | 0.750-0.929 |
| 3 | 791 | 9 | 0.983 | 0.005 | 0.974-0.992 | 51 | 3 | 0.786 | 0.051 | 0.692-0.892 |
| 4 | *No change* | | | | | | | | | |
| 5 | 576 | 11 | 0.964 | 0.007 | 0.950-0.978 | 45 | 3 | 0.734 | 0.056 | 0.632-0.851 |
| 6 | 460 | 9 | 0.945 | 0.009 | 0.927-0.964 | 41 | 2 | 0.698 | 0.059 | 0.592-0.822 |
| 7 | 383 | 15 | 0.908 | 0.013 | 0.883-0.934 | 36 | 1 | 0.678 | 0.060 | 0.570-0.807 |
| 8 | 186 | 4 | 0.889 | 0.016 | 0.858-0.921 |  |  |  |  |  |
| 9 | 88 | 1 | 0.879 | 0.019 | 0.843-0.916 |  |  |  |  |  |

**Weather effects**

We tested interactions between feather development, body condition index, and mass of Brewer’s sparrows and minimum temperature between relocations to examine whether the relative importance of these traits for thermoregulation could vary depending on the temperature. We did not test interactions for sagebrush sparrows or sage thrashers because of insufficient sample size. We did not include interactions in the final model suite because interactions did not improve model fit. Temperature did not affect the relative influence of morphology on exposure-caused mortality.


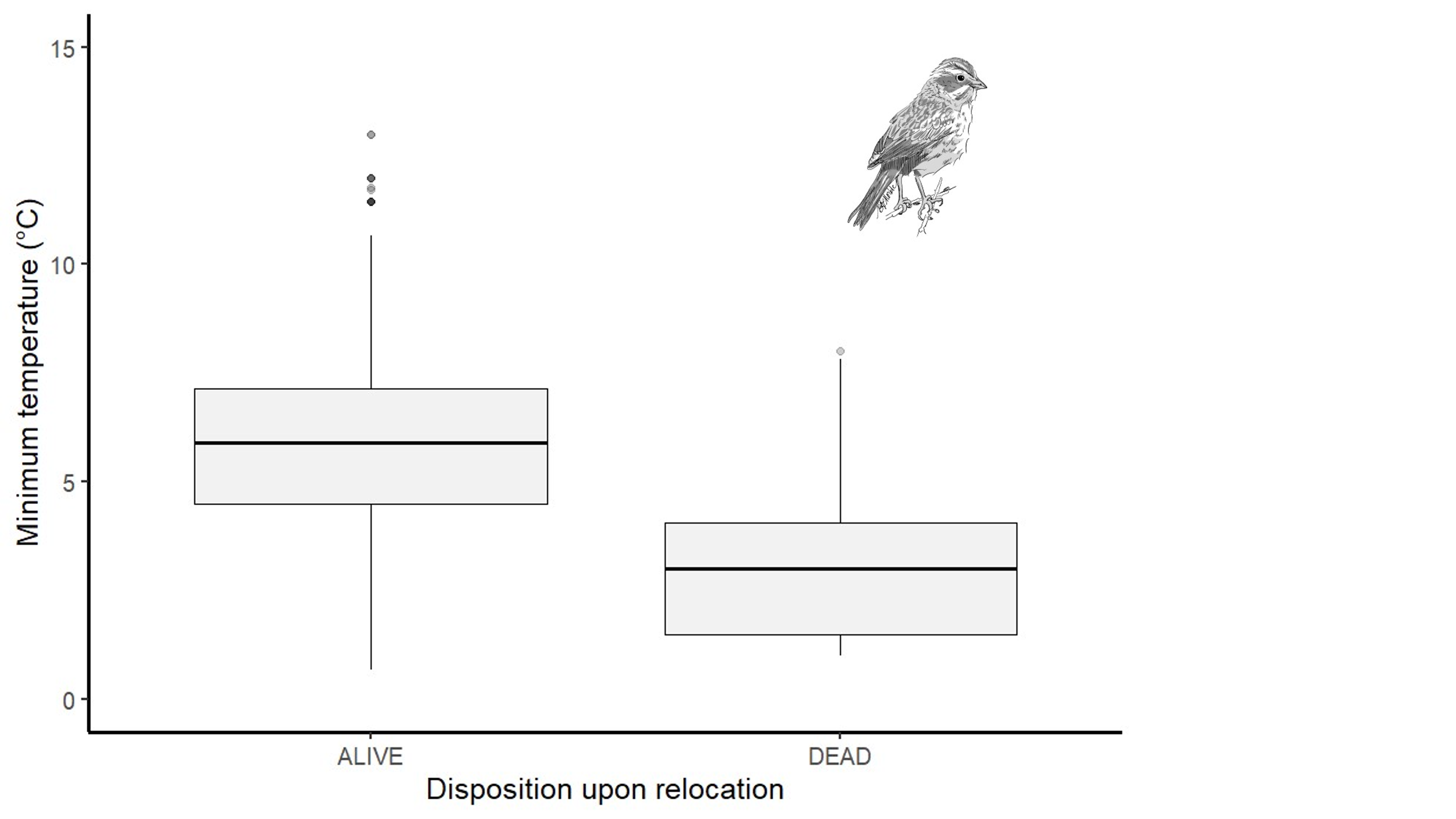
**Fig. S5** The minimum regional temperatures experienced by Brewer’s sparrow fledglings between relocations for fledglings that were alive at relocation were greater than temperatures experienced by fledglings that had died of exposure by the time of relocation. Fledglings were tracked in 2021 – 2023 in Wyoming, USA.
